# Primary Squamous Cell Carcinoma of the Thyroid Diagnosed as Anaplastic Carcinoma: Failure in Fine-Needle Aspiration Cytology?

**DOI:** 10.1155/2014/301780

**Published:** 2014-09-09

**Authors:** Fernanda Bolfi, Maria A. C. Domingues, Manuel Sobrinho-Simões, Paula Soares, Ricardo Celestino, Emanuel C. Castilho, Guareide Carelli, Norberto S. Paes, Glaucia M. F. S. Mazeto

**Affiliations:** ^1^Division of Endocrinology and Metabolism, Internal Medicine Department, Botucatu Medical School, São Paulo State University (UNESP), 18618-000 Botucatu, SP, Brazil; ^2^Pathology Department, Botucatu Medical School, São Paulo State University (UNESP), 18618-000 Botucatu, SP, Brazil; ^3^Institute of Molecular Pathology and Immunology of the University of Porto (IPATIMUP), 4200-465 Porto, Portugal; ^4^Head and Neck Department, Botucatu Medical School, São Paulo State University (UNESP), 18618-000 Botucatu, SP, Brazil; ^5^Division of Clinical Oncology, Internal Medicine Department, Botucatu Medical School, São Paulo State University (UNESP), 18618-000 Botucatu, SP, Brazil; ^6^Division of Radiotherapy, Department of Dermatology and Radiotherapy, Botucatu Medical School, São Paulo State University (UNESP), 18618-000 Botucatu, SP, Brazil; ^7^Division of Endocrinology and Metabolism, Internal Medicine Department, Botucatu Medical School (FMB), São Paulo State University (UNESP), 18618-000 Botucatu, SP, Brazil

## Abstract

A case of primary squamous-cell carcinoma (SCC) of the thyroid which had been initially diagnosed as an anaplastic carcinoma (ATC) is described: female, 73 years old, with a fast-growing cervical nodule on the left side and hoarseness for 3 months. Ultrasonography showed a 4.5 cm solid nodule. FNA was compatible with poorly differentiated carcinoma with immunoreactivity for AE1/AE3, EMA. Thyroidectomy was performed. Histopathological examination showed a nonencapsulated tumor. Immunohistochemistry disclosed positivity for AE1/AE3, p53,p63, and Ki67. The diagnosis was ATC. A second opinion reported tumor consisting of squamous cells, with intense inflammatory infiltrate both in tumor and in the adjacent thyroid, with final diagnosis of SCC, associated with Hashimoto thyroiditis. No other primary focus of SCC was found. Patient has shown a 48-month survival period. Clinically, primary SCCs of the thyroid and ATCs are similar. The distinction is often difficult particularly when based on the cytological analysis of FNA material.

## 1. Introduction

Squamous cell carcinomas (SCCs) of the thyroid are lesions consisting entirely of tumor cells with squamous differentiation [1]. Primary SCC is an extremely rare neoplasm, representing from 0.2% to 1.1% of all malignant tumors of the thyroid [2–5], with fewer than 100 cases described in the literature [6].

Here we report a rare case of primary SCC of the thyroid gland, which was initially diagnosed as anaplastic carcinoma (ATC), and we discuss the possible diagnostic difficulties.

## 2. Case Report

A 73-year-old woman, smoker, visited the endocrinology service in May 2010 with a 3-month history of a rapidly growing, adherent cervical nodule on the left side and hoarseness. Subsequent laboratory tests showed normal serum concentrations of thyrotropin (TSH), free thyroxine (FT4), calcitonin, and carcinoembryonic antigen (CEA) and increased levels of antiperoxidase antibodies (TPO Ab) (Table 1). Cervical ultrasonography (US) demonstrated a large, predominantly hypoechoic, heterogeneous nodule with well-defined boundaries and with central blood flow measuring 3.5 × 3.6 × 4.5 cm, in the left lobe of the thyroid (LTL). Fine-needle aspiration cytology of the mass was compatible with a poorly differentiated carcinoma that was probably anaplastic. Immunocytochemistry was positive for AE1/AE3 and focal EMA expression; negative for calcitonin, desmin, TTF-1, and p53 expression; and inconclusive for enolase, thyroglobulin, and synaptophysin expression.

An otorhinolaryngological examination found no signs of local invasion. Cervical nuclear magnetic resonance imaging revealed a 4.2 cm mass in the LTL, which compressed the adjacent structures and was isointense with thyroid parenchyma. Thoracic radiography showed widening of the upper mediastinum to the left and slight deviance of the trachea to the right, without pulmonary changes. The patient was referred for total thyroidectomy in June 2010, requiring tracheostomy.

**Figure 1:**
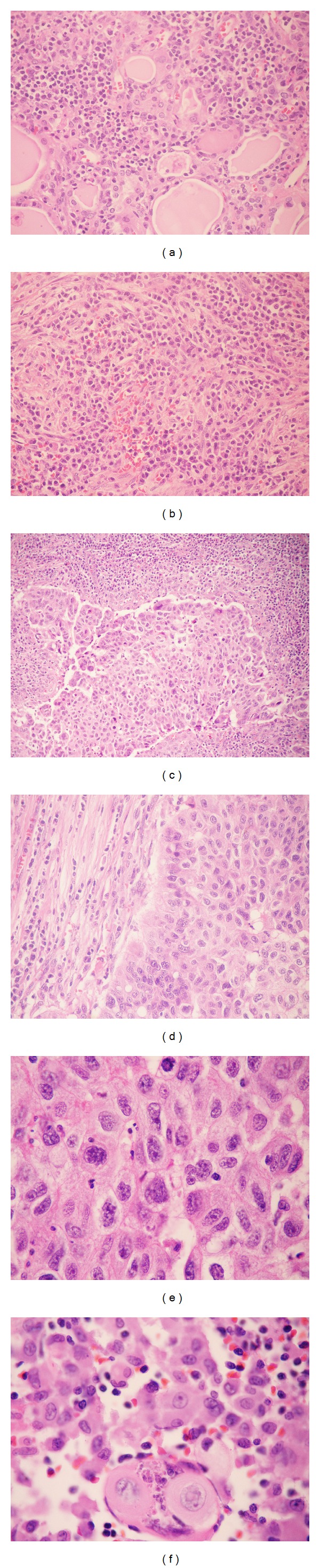
(a) Thyroid adjacent to the tumor with morphological appearance of Hashimoto's thyroiditis; follicles are surrounded by lymphoplasmacytic inflammatory infiltrate (40x, hematoxylin and eosin (HE)). (b) Fibrotic bands and inflammatory infiltrate (40x, HE). (c) Moderately differentiated squamous cell carcinoma (grade II) infiltrating the stroma of the thyroid (20x, HE). (d) Interface between squamous cell carcinoma and inflammatory stroma (40x, HE). ((e) and (f)) Squamous cell carcinoma showing cellular pleomorphism, bizarre nuclei, and keratinization of individual cells (1000x, HE).

**Figure 2:**
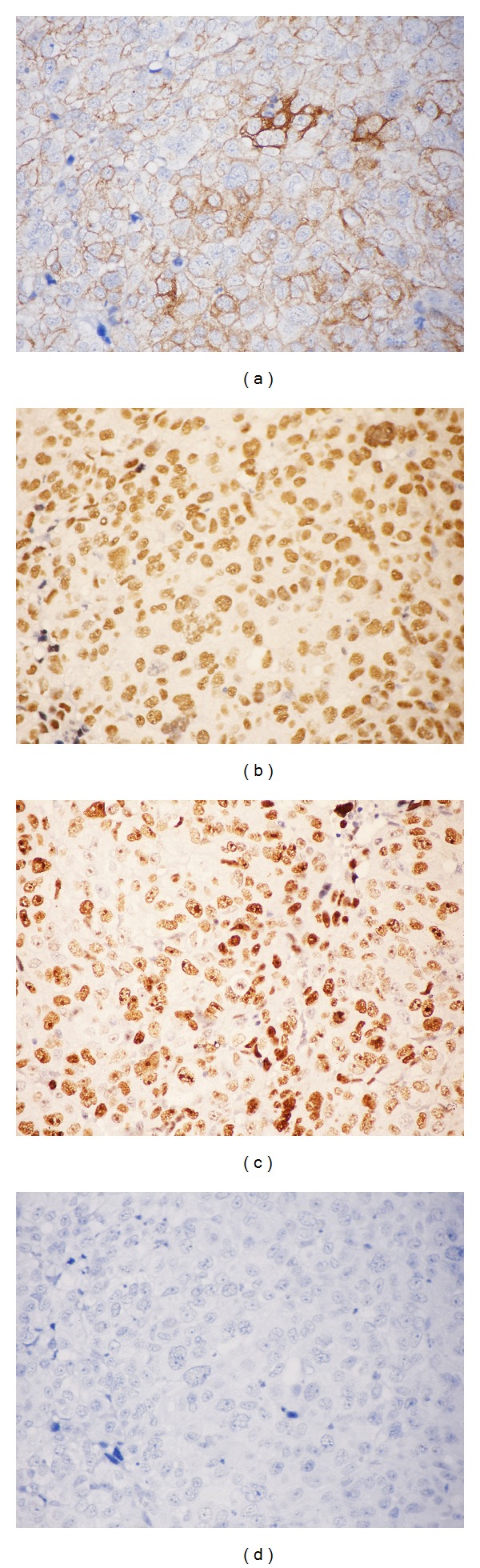
Immunohistochemical staining (IHC) evaluation of the tumor. (a) AE1/AE3: positive and diffuse cytoplasmic immunoexpression (400x); (b) p63: positive and diffuse nuclear immunoexpression (400x); (c) Ki-67: high cell proliferation index (approximately 70%) (400x); (d) thyroglobulin, TTF-1, CEA, and calcitonin: negative immunoexpression (400x).

Histopathological examination showed a 2.8 cm nonencapsulated tumor that occupied the left lobe and had a compromised left margin, extending up to subcutaneous soft tissues and trachea; no lymph nodes were resected (pT4aNxMx); HT was seen in adjacent tissue (Figure 1). Immunohistochemistry was positive for AE1/AE3, p53 (diffuse), p63, and Ki-67 (70%) expression and negative for CEA, thyroglobulin, TTF-1, and calcitonin expression (Figure 2). The tumor was diagnosed as probable ATC. However, because of the squamous aspect of the tumor, the possibility of SCC was suggested. The material was then submitted to second opinion consultation, and the findings were of a tumor predominantly composed of squamous cells and accompanied by intense inflammatory infiltrate of the adjacent thyroid and with chronic characteristics, of a fibrosing character, with vascular walls thickened by chronic inflammation secondary to HT. When the histological findings and previous immunohistochemical results were taken into consideration, the final diagnosis was SCC associated with active HT, thus excluding the diagnosis of ATC.

Additional work-up, including US of the abdomen, laryngoscopy, upper digestive endoscopy with biopsy of the gastric mucosa, mammography, and bone scintigraphy, did not reveal any other primary focus of SCC. The patient was referred for chemotherapy using Adriamycin and cisplatin and radiotherapy for a total dose of 5000 cGy (20 treatments × 250 cGy). To date, the patient has survived 48 months following resection of the tumor, with no evidence of recurrence.

## 3. Discussion

SCC of the thyroid gland can develop as primary or secondary disease. Secondary SCC is 10-fold more frequent than primary SCC and it is a result either of direct invasion by SCC from adjacent structures or of metastasis from distant sites to the thyroid [7–9]. We have described a patient with primary SCC of the thyroid, with inflammation and fibrotic destruction of thyroid tissue, which was initially diagnosed as ATC.

Primary SCC of the thyroid mainly affects female patients in their fifth and sixth decades of life, although there have been reports of younger patients with the disease in association with HT [10]. Upon presentation, the signs and symptoms include a history of sudden appearance of massive enlargement of the thyroid gland with or without cervical lymphadenopathy, associated with compressive complaints caused by infiltration of adjacent structures. At diagnosis, these tumors are generally found to be invading the trachea, esophagus, or large cervical vessels [11]. A definitive diagnosis of primary SCC of the thyroid, though being not easy, is crucial, since its treatment and prognosis differ from those of other thyroid neoplasms.

In this patient, the preliminary diagnosis was ATC. Clinically, primary SCCs of the thyroid and ATC are similar because they are both very aggressive [12]. Indeed, differentiation between these two types of lesions is often difficult, particularly when attempting diagnosis from fine-needle aspiration cytology, and additional differential diagnosis includes ATC with focal SCC component or squamous metaplasia [13] and the intrathyroid epithelial thymoma (ITET)/carcinoma showing thymus-like differentiation (CASTLE) [14]. ITET/CASTLE has less aggressive biological characteristics than primary thyroid SCC and the main features for the differential diagnosis are the positive immunoreactivity for CD5 and a proliferation index usually smaller than 20% [14]. In this patient, a definitive diagnosis was made after histological and immunohistochemical analysis of the resected lesion, which showed typical and diffuse tissue morphology of SCC, with a Ki-67 proliferation index of approximately 70%. This high proliferation index and the overexpression of p53 oncoprotein are associated with undifferentiated tumors and bad prognosis [10]. Negativity for TTF-1 and thyroglobulin expression, which are markers for follicular and papillary carcinomas [15], as well as for calcitonin, a marker of medullary thyroid carcinoma, excluded the possibility of these tumors, more commonly found in the thyroid.

Exclusion of primary lesions in other organs is performed to differentiate between primary and secondary SCC [9]. In this patient, no other primary foci of squamous cell lesions were found. In addition to distant primary lesions, local malignant transformation in an embryonic remnant or malformation (branchial or laryngeal clefts) can be the source of SCC [16]. In our patient, careful analysis of the entire representative sample of resected specimen did not reveal any evidence of this type of tissue, thus confirming the diagnosis of primary SCC of the thyroid.

Another interesting feature of this case was the intense background of Hashimoto's thyroiditis, showing an inflammatory infiltrate and a fibrosing pattern. In addition, there was intense chronic inflammation that resulted in thickened walls of blood vessels. Though being rare, the association between primary SCC of the thyroid and chronic thyroiditis has been found, and there are few reported cases [9, 13, 17–19]. In this context, SCC cells would originate from squamous metaplasia of follicular cells, which is promoted by chronic inflammation [7, 16].

The best treatment for SCC of the thyroid is early diagnosis followed by aggressive surgery, in order to prevent suffocation by bleeding or obstruction [12]. Adjuvant treatment combining chemotherapy with radiotherapy, in an attempt to reduce the risk of locoregional recurrence, has shown poor efficacy. Therefore, the prognosis of this malignancy is guarded, and in most cases, death occurs within 1 year [6]. Our patient underwent extensive surgery, radiotherapy, and chemotherapy, and she has shown no evidence of disease since the surgical procedure. The increased survival reported here suggests that a more “curative” surgery was possible in this case, which could explain a different pattern from that reported in the literature.

## Figures and Tables

**Table 1:** Preoperative laboratory findings (serum concentrations).

Tests	Value
TSH	4.67 uIU/mL (0.35–4.94 uIU/mL)
FT4	1.24 ng/dL (0.7–1.48 ng/dL)
TPO Ab	811 UI/mL (<5.61 UI/mL)
CEA	3.76 ng/mL (>5 ng/mL)
Calcitonin	<2.0 pg/mL (<11.5 pg/mL)

CEA: carcinoembryonic antigen; FT4: free thyroxine; TPO Ab: thyroid peroxidase antibody; TSH: thyroid stimulating hormone.
